# Can abuse deterrent formulations make a difference? Expectation and speculation

**DOI:** 10.1186/1477-7517-6-8

**Published:** 2009-05-29

**Authors:** Simon H Budman, Jill M Grimes Serrano, Stephen F Butler

**Affiliations:** 1 Inflexxion, Inc 320 Needham St Suite 100, Newton, Massachusetts 02464, USA

## Abstract

It is critical that issues surrounding the abuse and misuse of prescription opioids be balanced with the need for these medications for the treatment of pain. One way to decrease the abuse of prescription opioid medications is to develop abuse deterrent formulations (or ADFs) that in some way prevent drug abusers from extracting out the active ingredient in order to employ alternate routes of administration, such as injection, snorting, and smoking. Several factors including the pharmacokinetic profile of the drug, the features of the drug formulation that make it attractive or unattractive for abuse, the type of drug abuser, the progression of one's addiction pathway, and one's social environment may all play a role in the abuse of prescription opioids and what methods are used to abuse these drugs. This paper will examine these factors in order to understand how they affect the abuse of prescription opioids and routes of administration, and how the development of ADFs may alter these patterns.

## Introduction

The use of opioids for the management of chronic cancer pain and for palliative care is generally accepted in today's society, as is the use of opioids for moderate to moderate to severe acute pain. Prescription opioid medications are extremely effective in managing various types of pain; nonetheless, the use of opioids to treat chronic non-cancer pain remains controversial mainly due to the potential for negative impact on the patients, including abuse, dependence, and tolerance of these substances, leading to the under treatment of pain in many populations. Approximately 9% of Americans suffer from chronic, non-cancer-related pain [1]. Undertreated chronic non-cancer pain causes significant economic, societal, and health impacts [2], and may be related to a lack of education for physicians about proper prescribing practices and/or fear of being prosecuted as a result of one's prescribing patterns.

A 2006 National survey on prescription opioid abuse estimated that 5.2 million people in the United States used pain relievers nonmedically in 2006 and for the first time, prescription opioids surpasses marijuana as the drug most often associated with drug initiation [3]. A recent study on healthcare impacts resulting from substance abuse found that opioid abusers were 11.2 times more likely to have had at least one mental health outpatient visit and 12.2 times more likely to have had at least one hospital inpatient stay than non-abusers and were four times more likely to have had an emergency room visit than non-abusers [4]. An intricate balance is crucial between making prescription opioid medications available for appropriate candidates in pain and preventing these medications from being diverted for abuse.

Given the potential of abuse, pressure has been growing on pharmaceutical companies to develop prescription opioid formulations that, in some way, deter abuse and yet, remain readily accessible for pain management. For purposes of this manuscript, the term abuse refers to "the intentional self-administration of a medication for a non-medical purpose, such as altering one's state of consciousness, e.g. "getting high" [5], whereas the term misuserefers to "the use of a medication (for a medical purpose) other than as directed or as indicated, whether willful or unintentional, and whether harm results or not" [5]. It is hoped that the development of abuse deterrent formulations (ADFs) will decrease levels of abuse of prescription opioid medications. Various types of ADFs are currently being developed, each with a unique mechanism to thwart abusers' attempts to manipulate the drug so that the active ingredient is immediately available (especially for extended release formulations) in a form conducive for use via alternate routes of administration. Some new formulations that aim to reduce abuse through preventing alternate routes of administration employ physical barriers that resist common methods of tampering, which include crushing the pill, and subjecting the pill to various chemical manipulations in order to extract the active ingredient with the goal of preventing abuse through intravenous, snorting, and chewing routes of administration; this type of ADF, however, would not prevent abuse of the drug if the formulation is taken intact [6]. Antagonist-agonist combinations include an antagonist that blocks the effect of the opioid if it were to be tampered with, however, some studies have indicated that one such formulation (Talwin^® ^NX; Sanofi-Aventis, Bridgewater, New Jersey, USA) showed decreased efficacy in managing moderate pain [7]. Although ADFs are developed with the goal of decreasing abuse of prescription opioids though alternate routes of administration, they will likely have little to no impact on those who prefer to abuse these drugs by taking the drug intact. Other drug formulations being developed with the goal of deterring abuse include prodrugs, which have to be metabolized to an active form upon ingestion to produce a pharmacological effect and those that incorporate an aversive stimulus, such as niacin or capsaicin, which produces an uncomfortable physical sensation in the taker if the product is tampered with prior to ingestion [6]. It is clear that the maximum impact of these ADFs will most likely not be seen until, at the very least, most of the opioid analgesics prescribed are ADFs.

## What are drug abusers looking for in a prescription opioid?

The abuse potential of a drug is partially dependent upon the pharmacokinetic profile of the drug, including the chosen route of administration of the drug, how much of the drug is administered, and the rate of onset of its effects [8]. It is likely that the way in which abusers may be most affected by ADFs is in regard to the route(s) of administration that are used to "get high" with a particular drug. Routes of administration vary in the time it takes for the drug to reach the brain; it is believed that routes of drug administration that allow for a more rapid delivery are associated with greater abuse liability. For the majority of drugs, including opiates, routes of administration can be ranked from fastest delivery method to slowest as follows: inhalation (i.e. smoking), intravenous, intranasal, and oral [9], although most opioids are well absorbed through all routes of administration. Jenkins et al. (1994) showed that although smoking heroin and administering heroin intravenously produced detectable levels of heroin in the blood after 1–2 minutes at similar doses, smoking produced lower blood levels than was observed for intravenous administration [10]. Mansbach and colleagues (2006) [11] indicate that: "most of the research supports the hypothesis that a rapid rate of rise in plasma concentration is more likely to result in drug liking and reinforcement than a slower rise in plasma exposure" (p. S16). The time required for a given drug, at a specific dose and route of administration (Tmax) to achieve peak plasma concentrations (i.e. when Cmax is achieved) is directly related to the reinforcement properties of the drug. Research in both animals and humans has, in fact, shown that faster infusion rates of many drugs of abuse, including cocaine [12], nicotine [13], sedatives (i.e. pentobarbital and diazepam) [14,15], and morphine [11], which results in higher plasma levels of the drug, produced increased response rates in animals and greater positive subjective effects (i.e. "high" and drug craving) in humans than slower infusion rates. It is likely this increase in drug blood plasma levels (Cmax) results in the "high" or "rush" that some drug abusers seek, possibly resulting in an increased abuse liability of the drug.

Aside from achieving rapid delivery and/or maximum concentrations of the opioid's active ingredient in the brain, there are a number of other factors, including how attractive the opioid formulation is to potential abusers and the length of time one has been abusing opioids, for instance, that also contribute to the perceived attractiveness of a given opioid, which will be described in full detail in the following sections.

## The concept of opioid attractiveness

Attractiveness of a particular drug formulation may influence the extent to which a drug is abused. Previous research has characterized the potential attractiveness of opioids via examination of the drug's time to onset; the method of administration, and maximum plasma concentrations following administration [16,17] while others have attempted to systematically examine how perceived qualities and features of formulations contribute to a specific opioid product being viewed as more or less attractive to those with a history of misusing/abusing this class of drugs [18]. In this latter study, 10 factors most related to the attractiveness of prescription opioid formulations were identified and significant differences in the weights attributed to the features and the corresponding factors could be observed between those who prefer different routes of administration (e.g., swallowing, chewing or sucking (buccal), snorting or smoking, and injecting). Furthermore, significant differences were found between the different groups (which varied on preferred route of administration) and that the model fit well for those who preferred alternate routes of administration (i.e. injectors, snorters, and smokers) and not for those who preferred to take prescription opioids by swallowing. Thus, the new abuse deterrent formulations that alter the physical and/or chemical properties of the drug to prevent extraction may be most likely to impact the attractiveness of that drug for those who snort, smoke, or inject prescription opioids.

**Figure 1:**
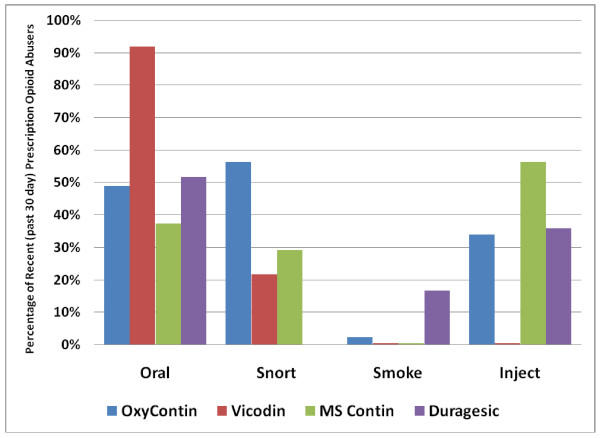
**Routes of Administration for Various Prescription Opioids**.

Research using data from the ASI-MV^® ^Connect Component of the NAVIPPRO™ system [19] also indicates that prescription opioids appear to have "typical" patterns of route of administration employed by those who abuse these drugs and are in treatment for their substance abuse problems. That is, some drugs, such as Vicodin^® ^or Percocet^®^, are almost never injected, whereas other drugs, such as morphine sulfate (e.g. MS Contin^®^, Kadian^®^, and Avinza^®^), have a high rate of injection among those who abuse them. OxyContin^®^, has a more "versatile" routes of administration profile in that it is likely to be abused through a variety of different routes as was observed in a population of individuals seeking substance abuse treatment who indicated past 30 day abuse of prescription opioids (N = 4,807) at various substance abuse treatment centers throughout the United States (Figure 1). Briefly, participants for this study comprised of clients 18 years and older attending substance abuse treatment centers across the United States who completed the ASI-MV^® ^Connect as part of their treatment experience. The ASI-MV^® ^Connect is purchased by treatment facilities for efficient and cost-effective patient evaluation and treatment planning purposes and is used as part of the standard clinical intake to measure patients' medical, employment, drug, legal, family and social relationships, and psychiatric problems. For purposes of the data presented here, prescription opioid use was operationalized as self-reported past 30-day use of any prescription opioid while prescription opioid abuse was operationalized as self-reported past 30-day use of any prescription opioid "in a way not prescribed by your doctor, that is, taking it for the way it makes you feel and not for pain relief". For full details on the sample and methods for this study, please refer to [19].

It is clear that the route of administration profile of a drug is very important in how some abusers view a particular drug formulation and in patterns of abuse that are observed. It is likely that it is an interplay of a variety of factors that determine what routes of administration one chooses when abusing certain drugs and that the development of ADFs may change the patterns of behavior associated with prescription opioid abuse by making the active ingredient less accessible and, therefore, less attractive to those who prefer to abuse these drugs via alternate routes of administration.

## Different types of prescription opioid abusers and routes of administration

It is also important to note that there are different types of prescription opioid abusers. In a seminal study, Green and colleagues studied prescription substance abusers entering treatment using data from the NAVIPPRO™ system [20]. Applying latent class analysis to this data, six classes were identified as clinically interpretable and relevant subgroups of prescription opioid abusers. These classes were labeled, based on their item-response probabilities and for discussion purposes, classified as: Prescribed Misusers; Healthy Abusers; Poly-prescription Opioid Abusers Who Inject; Poly-prescription Opioid Abusers Who Snort; OxyContin^® ^plus Heroin Abusers; and Methadone and Other Opioid Abusers. These classes were distinct in their prescription opioid abuse practices and preferences including drug preferences, reporting of pain problems, preferred routes of administration, and socio-demographic characteristics, among others. Three classes of prescription opioid abusers were identified as being injectors of opioids and/or illicit drugs and while all three classes were characterized as injecting prescription opioids and heroin, some differences exist. Poly-prescription opioid injectors injected other drugs, including cocaine and amphetamines, whereas those in the OxyContin^® ^plus Heroin group injected only heroin and used cocaine through non-intravenous means. Furthermore, while the Poly-prescription Opioid Injectors and the OxyContin^® ^+ Heroin Abusers were experienced prescription opioid abusers, those in the Methadone and Other Opioid Abuser group were newer to prescription opioid abuse. A stark comparison can be made between those who indicate alternate routes of administration when abusing prescription opioid and other drugs and those who do not, in that the former appear to be newer to prescription opioid abuse. Through the development of ADFs, it may be possible to influence certain types of prescription opioid abusers, particularly those who prefer to extract the active ingredient out of the drug formulation as to allow for alternate routes of administration.

## Natural history of opioid abuse

Another interesting component related to routes of administration is the natural history of prescription opioid abuse. In a cross-sectional study, Butler and colleagues explored the "natural history" of prescription opioid abuse in order to understand the different patterns that emerge as a function of the amount of time one has abused any opioid (i.e. prescription opioids, heroin, and/or methadone) and one's age upon seeking treatment for substance abuse, with respect to routes of administration, other types of drugs abused, and the presence of problems known to be associated with substance abuse dependence [21]. Results from this study showed that, overall, the longer one has abused any opioid, the more likely one is to use alternate routes of administration (i.e. injection and/or snorting); are more likely to abuse illicit drugs (lifetime and/or past 30 days); and are more likely to report problems in various areas of life functioning according to the Addiction Severity Index (ASI). This study also revealed that one's age also had a significant effect on various behaviors/outcomes of drug abuse; the younger adult population (i.e. not adolescent population) of those seeking substance abuse treatment was more likely to use alternate routes of administration, use illicit drugs, and have greater problem severity as measured by the ASI. Furthermore, an interaction between these two risk factors (length of abusing any opioid and age) appears to exist so that the length of time abusing any opioid and the younger one is the more likely one is to use alternate routes of administration (i.e. injection), abuse illicit drugs, and to experience psychological problems as measured by the ASI. These results indicate that, in this particular substance abuse treatment seeking population, the younger group of prescription opioid abusers may consist of high risk takers and the older prescription opioid abusers may be more risk averse. These results may be similar to other risk-related behaviors including those associated with high-risk sexual behaviors. Research has shown that a greater percentage of respondents in the younger population (aged 18–24 years) reported having more multiple sexual partners (21.3, range 17.0–26.4 versus 4.1, range 3.1–5.5) and had greater HIV-specific risk factors associated with them (i.e. intravenous drug abuse, treatment for sexually transmitted diseases during the preceding year, or a positive HIV test) (10.7, range 7.2–15.6 verses 2.6, range 1.7–4.0) than an older population (aged 35–44) [22]. A successful ADF would presumably prevent preparation of the drug formulation for injection and inhibit this commonly seen progression from abusing an opioid orally to injecting it.

## Routes of administration and social environment

Social environment appears to play an integral role in determining whether a non-injecting drug user initiates drug use via intravenous means. Research has discovered that non-injecting users with social networks that consist of injecting drug users (IDUs) are at a greater risk of injecting [23,24]. Although most non-injecting drug users initially express negative feelings toward injection practices, these feelings are modified by other factors including social pressures and being associated with a group of injectors; the lure or appeal of experimenting with injection; the notion that the drug has greater efficacy when it is injected, and the belief that one needs to use less of it in order to achieve the same "high" [24]. Not only do drug abusers learn about new routes of administration from other, more "experienced" drug abusers in their social circle, but the Internet offers a great deal of information on routes of administration, including step-by-step instructions on how to administer drugs through various routes, and may provide drug abusers with a discrete way of inquiring about alternate methods of drug administration [25]. Presumably, given a successful ADF, Internet communications about the drug may still persist, but the focus of the discussion may transition from efforts to alter the formulation for purposes of abuse to expressions of frustration about not being able to alter the formulation for alternate routes of administration.

## Drug availability

Throughout history the availability of a drug has been shown to influence its patterns of abuse. This was demonstrated particularly well during the heroin shortage in Australia in 2001. Researchers noted that heroin was the most frequently injected drug in Australia from 1996–2000, however, in 2001, a prolonged reduction in the availability of heroin and subsequent increase in cost occurred [26]. Not only did heroin IDUs shift their drug of choice to other drugs, particularly stimulants (i.e. cocaine and methamphetamine) without a change in preferred route of administration (i.e. heroin IDUs switched to injecting cocaine or methamphetamine) [26-28] but there was a drop in heroin-mediated overdoses [27] possibly due to a decrease in the rate of heroin injection in this population [26,28].

Research by Dasgupta et al (2008)[29] and Brownstein et al (Submitted) indicate that there is a direct relationship between the amount of a prescription opioid available for medical purposes in a given geographic area and its abuse in that area. It is presumably the case that even if it is available an abuser whose preferences are to inject or snort will not do so with Vicodin^® ^because of the presence of acetaminophen. Likewise, an abuser whose preference is to inject oxycodone will be unlikely to try to do so if the formulation he or she has available is not readily prepared for injection. Therefore, one could predict development of abuse deterrent opioid formulations would result in increases in legitimate prescribed availability and appropriate, medical use of opioid medications without a corresponding increase in reported abuse or injection of these formulations.

## Negative factors associated with illicit routes of administration

A critical factor in success of opioid ADFs appears to be strongly related to their ability to decrease illicit routes of administration, particularly intravenous drug abuse. Several studies have indicated that a number of negative factors may be associated with illicit routes of administration. Illicit routes of administration have been associated with poor interpersonal relationships, work performance, and legal problems [20]. Increased violence has been documented among IDUs [30] as well as increased rates of homelessness, leading to a variety of health and societal issues [31]. Furthermore, illicit routes of administration are linked to increased risk of non-fatal overdose [32-34], increased risk of mortality [35,36], and increased psychiatric comorbidity [37]. A recent report on unintentional pharmaceutical overdose fatalities in West Virginia showed that individuals who used diverted drugs were more likely to use a nonmedical route of administration and to have combined prescription with illicit drugs upon overdosing [38]. However, the increased risk of contracting and subsequently transmitting blood-borne diseases such as HIV, HVC, and HBV is particularly concerning among intravenous drug users [20,39-41]. It is possible that ADFs that can decrease alternate routes of administration, in particular intravenous routes, may positively impact the health and functioning of those abusing prescription opioids via alternate routes of administration, however, there still lies the possibility that intravenous drug abusers will simply seek other drugs (i.e. heroin) that are easily injected.

## A multi-component model for understanding prescription opioid abuse and routes of administration

What determines the chosen routes of administration for abusers of prescription opioids? Several factors including drug formulation, drug availability, the course of an individual's drug abuse history, one's social environment, and/or the availability of information on how to prepare a drug formulation for alternate routes of administration, may be highly correlated with one another and they appear to be some of the important determinants in what routes of administration a drug abuser chooses. The inverse may also be true, in that, the decisions one makes about routes of administration may influence or be influenced by the make-up of one's social network, what formulation characteristics are important (i.e. having the ability to extract the active ingredient from the drug formulation), and the experience of problems associated with drug abuse.

Formulation is only one of a number of important components that may be relevant to routes of administration. However, formulating a drug to make it more difficult for an abuser to use via an illicit route may impact which drugs an abuser chooses. It also follows that if certain preferred routes of administration are closed off to the potential abuser, that individual may choose to use an alternative product. In the ideal circumstance, all opioid products available for medical use would be very difficult to abuse via illicit routes of administration.

## Discussion

ADFs are unlikely to be a panacea. However, within a broader context they may well have positive public health effects. It is clear that a variety of factors help to determine which routes of administration are used by individual abusers. These factors may relate to properties of the drug itself and/or to other personal, interpersonal, and/or societal factors. To the degree that a formulation itself may help mitigate abuse, the ADFs could have an impact on rates of abuse of prescription opioids. It is certainly possible that if a particular ADF employs a mechanism that prevents abusers from crushing, dissolving, melting, etc., the actual pill, then certain routes of administration will likely be eliminated for that particular drug. However, there is no mechanism that is going to prevent abusers from taking the drug as it was meant to be taken in excess, and thereby still abusing the drug. The introduction of ADFs with a certain level of difficulty added to frustrate the extraction or tampering process may impede alternate or unintended routes of administration and therefore, may have benefits on public health in a variety of ways. A drug formulation that is able to prevent a certain route of administration (i.e. intravenous administration), that, in an historical context, has been responsible for the transmission of various blood-borne diseases, such as HIV, HCV, and HBV, may help to decrease the transmission rates of these illnesses. Furthermore, research has shown that intravenous drug abuse is associated with other risky behaviors such as polydrug abuse [42] and may indicate a greater severity of drug dependence [43-45]. If an ADF can be effective in making it more difficult to abuse the drug using alternate routes of administration, a significant societal impact may include decreased healthcare costs associated with drug abuse treatment.

In any case, it is unlikely that drug formulation alone will be sufficient to address prescription opioid misuse, abuse, and addiction. Educational and preventive interventions, for both patients and clinicians, will continue to play an important role in ultimately lessening the abuse of prescription opioids. Finally, the overall impact of abuse deterrent formulations will need to await long-term epidemiological studies which can track the overall impact of these drugs in comparison with other similar products without such safeguards. It is only with such careful scientific evaluation that we will learn the actual real world impact of this new class of drugs. Nonetheless, it is conceivable that ADFs can be helpful to overall efforts to reduce prescription drug abuse and can play a valuable role in broad scale programs focused on achieving such reductions.

## Competing interests

The writing of this paper was funded in part by King Pharmaceuticals, Inc. The views expressed in this paper are those of the authors and do not necessarily represent the views of King. The authors had sole editorial rights over the manuscript.

## Authors' contributions

All authors contributed to conceptualization, design, and write-up. All authors read and approved the final manuscript.
